# The impact of workplace violence on the mental health of health care workers during the COVID-19 pandemic

**DOI:** 10.47626/1679-4435-2023-1152

**Published:** 2024-09-24

**Authors:** Larissa Harada, Renata de Melo Branco, Thamires Ferreira Barbosa, Dinarte Ballester, Maria Tavares Cavalcanti, Andrea Tenorio Correia da Silva

**Affiliations:** 1 Faculdade de Medicina de Jundiaí (FMJ), Jundiaí, SP, Brazil; 2 Faculdade Santa Marcelina, São Paulo, SP, Brazil; 3 Universidade Federal do Rio Grande, Rio Grande, RS, Brazil; 4 Universidade Federal do Rio de Janeiro, Rio de Janeiro, RJ, Brazil; 5 Departamento de Saúde Coletiva, FMJ, Jundiaí, SP, Brazil

**Keywords:** COVID-19, violence, health personnel, mental health, covid-19, violência, pessoal de saúde, saúde mental

## Abstract

**Introduction:**

The COVID-19 pandemic introduced a new set ofwork-related stressors for health care workers.

**Objectives:**

This study aimed to investigate the associations between exposure to violence and common mental disorders among health care workers in emergency care settings during COVID-19 in the city of São Paulo, Brazil.

**Methods:**

We randomly selected two emergency care units. The 12-item General Health Questionnaire was used to assess common mental disorders among emergency health care workers (n = 100). We examined the relationships between common mental disorders and COVID-19 pandemic-related variables, including availability of personal protective equipment, exposure to violence, discrimination, harassment, and confidence in the workplace to handle the pandemic. We used multivariate Poisson regression with robust variance to estimate prevalence ratios for common mental disorders.

**Results:**

Overall, 50% (95%CI 39.8-60.1) of participants had a common mental disorder. In addition, 71% reported being victims of at least one type of violence during the COVID-19 pandemic. Higher risks ofcommon mental disorders were found among those who reported lacking personal protective equipment, being victims of discrimination, violence, or harassment, and reporting less confidence in the workplace to handle the pandemic. Participants exposed to two types of violence and three types of violence had higher prevalence ratios, with prevalence ratios of 2.28 (95%CI 1.23–4.21) and 3.14 (95%CI 1.62–6.08), respectively.

**Conclusions:**

Promoting access to personal protective equipment, addressing mistreatment of health workers as well as promoting their well-being at work, and building confidence in the workplace to deal with the pandemic are critical.

## Abstract (pt)

**Introdução:**

A pandemia de covid-19 trouxe uma nova gama de estressores relacionados ao trabalho para os profissionais de saúde.

**Objetivos:**

Este estudo examina as associações entre a exposição à violência e os transtornos mentais comuns entre os profissionais de saúde durante as situações de emergência na pandemia de covid-19 em São Paulo, Brasil.

**Métodos:**

Selecionamos aleatoriamente dois serviços de emergência. O Questionário Geral de Saúde 12 foi utilizado para avaliar os transtornos mentais comuns entre os trabalhadores de saúde de urgência (n = 100). Investigamos as relações entre transtornos mentais comuns e variáveis relacionadas com a pandemia: disponibilidade de equipamento de proteção pessoal, exposição à violência/discriminação/assédio e nível de confiança no local de trabalho para lidar com a pandemia. Realizamos uma regressão multivariada de Poisson com uma variação robusta para estimar as razões de prevalência para transtornos mentais comuns.

**Resultados:**

Entre os participantes, 50% (intervalo de confiança de 95% [IC95%] 39,8-60,1) apresentaram transtornos mentais comuns e 71% relataram ter sido vítimas de pelo menos um tipo de violência durante a pandemia de covid-19. Foi encontrado maior risco de transtornos mentais comuns entre aqueles que reportaram falta de equipamento de proteção pessoal, sendo vítimas de discriminação, violência ou assédio; e que reportaram menor confiança no local de trabalho para lidar com a pandemia. Os participantes expostos a dois tipos e a três tipos de violência apresentaram razão de prevalência 2,28 (IC95% 1,23-4,21) e razão de prevalência 3,14 (IC95% 1,62-6,08) mais elevados, respectivamente. Os resultados indicam domínios que podem ser cruciais para mitigar os transtornos mentais comuns entre os trabalhadores da saúde.

**Conclusões:**

É crucial promover o acesso a equipamento de proteção pessoal, combater os maus tratos e promover o bem-estar e confiança no local de trabalho para lidar com a pandemia.

## INTRODUCTION

The COVID-19 pandemic introduced a new set of work-related stressors for health care workers (HCWs).^1^ The high transmissibility of the severe acute respiratory syndrome coronavirus (SARS-CoV-2) led to an exponential increase in cases in a short period of time, resulting in overcrowding in health facilities and work overload due to the high number of patients requiring treatment and the need to cover for colleagues who were sick with COVID-19 and unable to work. These stressors were compounded by the lack of personal protective equipment (PPE), the novelty of the disease, the fact that patients could deteriorate and die quickly, the depletion of health system resources, the need to decide which patients would receive limited resources, and rapidly changing protocols.^2,3^

The prevalence of depressive symptoms and anxiety among HCWs during the pandemic ranged from 17.9% to 36% and 22.2% to 33%, respectively.^4^ However, few studies have assessed the mental health of HCWs during the COVID-19 pandemic in Brazil. In addition, studies have rarely examined the relationship between exposure to violence, discrimination, and harassment and the mental health of HCWs during the pandemic.

Prior to the COVID-19 pandemic, exposure to workplace violence was associated with depression among HCWs in the city of São Paulo. Among the 2,940 HCWs who participated in the PANDORA-SP study,^5^ 44.9% of them reported being insulted, 24.5% reported being threatened, 2.3% were victims of physical violence, and 29.5% witnessed violent behavior toward their colleagues. Physicians had a higher risk of being threatened, while community health workers (CHWs) were more likely to witness violence at work. The risk of depression was higher among professionals who reported exposure to violence.

In the context of COVID-19 pandemic, few studies have documented the incidence of violence against HCWs. As of July 2020, 265 COVID-19-related attacks on HCWs were reported in 61 countries.^6^ The types of violence experienced by HCWs included threats, verbal aggression and physical aggression.^7^ Some factors that exacerbated violence against these workers during this period were social isolation measures, limited access to health care, ineffective administrative policies, and the large number of deaths due to COVID-19, which caused fear, anxiety, irritation, and panic among the population.^8^

In addition, misinformation and out-of-context quotes on social media about the spread of the SARS-CoV-2 virus exacerbated the situation by stigmatizing HCWs as carriers of the virus.^8^ This increase in violence was harmful because violence is associated with reduced quality of life^9^ and increased rates of anxiety, depression, insomnia, self-harm, and suicide.^10^ Thus, violence adds to the psychological distress of already overburdened HCWs and increases the risk of common mental disorders (CMDs).^11^ Violence also compromises the job quality of HCWs and is associated with increased rates of absenteeism and burnout.^12,13^

Brazil is one of the epicenters of the COVID-19 pandemic, and the city of São Paulo is a major epicenter within Brazil in terms of both cases and deaths.^14^ In addition, as the largest urban center in South America, São Paulo has a high rate of urban violence, which intensified during the pandemic, especially against HCWs. We assessed CMDs among HCWs in emergency care units in São Paulo and examined the relationships between COVID-19 pandemic-related factors (access to PPE, exposure to discrimination, violence, and harassment) and mental health of HCWs.

## METHODS

### STUDY DESIGN AND PARTICIPANTS

We analyzed baseline data from the COVID-19 Healthcare Workers study in the city of São Paulo (HEROES-SP). HEROES-SP is a part of the HEROES study, a longitudinal global initiative involving 26 countries.^15^ We conducted an online survey from October to November 2020. Sample was obtained by randomly selecting two emergency care units in the 1st Region of São Paulo (n = 100). Response rate for the HEROES-SP study was 39.3%.

### MEASURES

The Spanish version of the HEROES questionnaire was translated into Portuguese and then back-translated according to World Health Organization standard procedures.^16^

### OUTCOME

CMDs were assessed by using the Brazilian version of General Health Questionnaire 12 (GHQ-12).^17^ This 12-item questionnaire assesses the presence of nonpsychotic mental disorders, particularly anxiety and depression, by asking whether the respondent has recently experienced certain symptoms and behaviors. Half of the items are worded positively (e.g., “During the past week, have you lost sleep due to being worried?”), while the other half are worded negatively (e.g., “During the past week, have you felt capable of making decisions?”). All items are rated on a 4-point Likert scale ranging from 0 (“not at all”/“much less than usual”) to 3 (“[much] more than usual”). Consistent with previous studies, including some from Brazil, we used a score of 3 or higher to indicate CMD.

### EXPOSURES

We assessed the following factors that were hypothesized to be associated with CMDs: (1) level of access to PPE (sufficient, a little insufficient, and much/very much insufficient); (2) experiences of mistreatment: discrimination (“I have felt discriminated due to being a health worker during the pandemic” [yes or no]), violence (“I have experienced violence due to being a health worker during the pandemic” [yes or no]), and harassment (“I have been harassed by family members of patients with COVID-19” [yes or no]); response options ranged from 0 to 3 types of violence; (3) job type (e.g., physician, nurse, nurse aide, administrative staff, etc.); (4) trust in workplace to handle COVID-19 pandemic ( none, a little, and a lot).

### CONFOUNDERS

We considered the following sociodemographic variables as potential confounders: age (18–30, 31–40, over 40 years), gender (female, male, other), and self-reported skin color (white, black, brown, etc.).

### STATISTICAL ANALYSIS

We used Poisson regression analyses with robust variance estimates to obtain prevalence ratios (PRs) for the associations between exposures and CMDs. Poisson regression was chosen to minimize overestimation of associations since the outcome was common in our sample.^18^ All analyses were performed by using Stata version 14.0 (StataCorp LP, College Station, TX).

### ETHICAL CONSIDERATIONS

The project was approved by the research ethics committee of the Municipal Health Secretariat of the city of São Paulo (registry number 4.160.385), by the research ethics committee of the Pan American Health Association (registry code PAHOERC.0208.02), and the National Research Ethics Commission (registry number 4.160.552). The ethics committee approval number is 33347120.3.3001.0086. All HCWs who were invited to participate in the study were informed of its objectives and procedures. Those who agreed to participate signed an informed consent form that guaranteed the confidentiality of their information and their right to withdraw consent at any time during the study.

HEROES uses a web-based platform to collect data across countries. This platform is similar to REDCap in terms of privacy and data management. To ensure confidentiality, each participant is assigned an identification number generated by a coding system. Access to the system is restricted to personnel with credentials defined by the study steering committee.

## RESULTS

**Figure 1:**
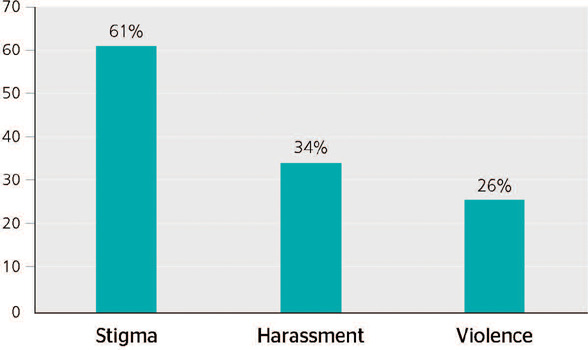
Type of mistreatment reported by health care workers during the COVID-19 pandemic (%).

Overall, 50% (95% CI 39.8-60.1) of HCWs presented with CMDs. Most participants were women (78%). Regarding COVID-19 pandemic-related factors, 43% of respondents had inadequate access to PPE; 50% reported a high level of trust in the workplace to deal with the pandemic; 71% reported experiencing at least one type of mistreatment (Table 1). Stigma was the most reported type of mistreatment (Figure 1).

**Table 1:** Distribution of participants by sociodemographic and work characteristics, exposure to violence in the COVID-19 pandemic, and CMDs. Data from HEROES study, São Paulo, Brazil

	Total n (%)	CMD	
		No n (%)	Yes n (%)
Sex			
Woman	78 (78.0)	39 (50.0)	39 (50.0)
Men	21 (21.0)	11 (52.4)	10 (47.6)
Age (years)			
18–30	35 (38.0)	18 (51.4)	17 (48.6)
31–40	40 (43.5)	20 (50.0)	20 (50.0)
Over 40	17 (18.5)	8 (47.1)	9 (52.9)
Skin color (self-reported)			
White	48 (48.0)	25 (52.1)	23 (47.9)
Black	16 (16.0)	8 (50.0)	8 (50.0)
Brown	32 (32.0)	16 (50.0)	16 (50.0)
Other	4 (4.0)	1 (25.0)	3 (75.0)
Job type			
Nurse aide	34 (34.0)	20 (58.8)	14 (41.2)
Nurse	15 (15.0)	6 (40.0)	9 (60.0)
Physician	4 (4.0)	1 (25.0)	3 (75.0)
Administration	31 (31.0)	13 (41.9)	18 (58.1)
Other*	16 (16.0)	10 (62.5)	6 (37.5)
Access to PPE			
Sufficient	57(57.0)	33 (57.9)	24 (42.1)
Insufficient	43 (43.0)	17 (39.5)	26 (60.5)
Trust in the workplace to handle the pandemic			
None	16 (16.0)	3 (18.7)	13 (81.3)
A little	34 (34.0)	14 (41.2)	20 (58.8)
A lot	50 (50.0)	33 (66.0)	17 (34.0)
Exposure to mistreatment			
None	29 (29.0)	18 (62.1)	11 (37.9)
One type	33 (33.0)	22 (66.7)	11 (33.3)
Two types	26 (26.0)	8 (30.7)	18 (69.3)
Three types	12 (12.0)	2 (16.7)	10 (83.3)

CMD = common mental disorders; PPE = personal protective equipment.

*Laboratory and X-ray technologists.

The variables associated with CMDs in professionals working in the Unidades de Pronto Atendimento (UPAs) were as follows: age group, participants aged over 40 years (adjusted PR = 1.89; 95%CI 1.01-3.55), degree of confidence in the ability of the workplace to cope with the COVID-19 pandemic (highest degree of confidence, adjusted PR = 0.43; 95%CI 0.24-0.74), and exposure to violence/discrimination as a result of being a health worker in the context of the COVID-19 pandemic or harassment by patients’ family members. The participants who reported exposure to more types of aggression had a higher odds ratio (OR) of CMD; thus, we observed that the risk was 2.28 higher for those who reported exposure to two types of aggression and three times higher for those who reported exposure to all three types (Table 2).

**Table 2:** Associations between participant characteristics, variables related to the context of the COVID-19 pandemic, and CMDs. Data from HEROES study, São Paulo, Brazil

	CMDs			
	Crude PR (95%CI)	p value	Adjusted PR (95%CI)	p value
Sex				
Women	1		1	
Men	1.00 (0.62–1.60)	1.000	1.11 (0.70–1.73)	0.640
Age (years)				
18–30	1		1	
30–40	1.14 (0.72–1.79)	0.900	1.24 (0.77–2.01)	0.360
40 or more	1.14 (0.66–1.95)	0.760	1.89 (1.01–3.55)	0.040
Skin color (self-reported)				
White	1		1	
Black	1.04 (0.58–1.85)	0.880	1.07 (0.61–1.88)	0.790
Brown	1.04 (0.66–1.64)	0.180	1.40 (0.85–2.30)	0.170
Other	1.56 (0.82–2.97)	0.170	1.63 (0.67–3.95)	0.270
Job type				
Nurse aide	1		1	
Nurse	1.45 (0.81–2.60)	0.200	1.60 (0.91–2.82)	0.100
Physician	1.82 (0.90–3.65)	0.090	1.80 (0.97–3.35)	0.060
Administration staff	1.41 (0.85–2.33)	0.180	1.67 (0.95–2.92)	0.070
Other*	0.91 (0.42–1.93)	0.800	1.15 (0.58–2.29)	0.670
Access to PPE				
Sufficient	1		1	
Insufficient	1.43 (0.97–2.12)	0.070	0.87 (0.54–1.42)	0.590
Trust in the workplace				
None	1		1	
Low	0.72 (0.50–1.04)	0.080	0.99 (0.57–1.74)	0.990
A lot	0.41 (0.26–0.65)	< 0.001	0.43 (0.24–0.74)	0.003
Exposure to discrimination/stigma and violence				
None	1		1	
One type	0.87 (0.44–1.72)	0.70	1.25 (0.61–2.53)	0.520
Two types	1.82 (1.06–3.11)	0.02	2.28 (1.23–4.21)	0.008
Three types	2.19 (1.28–3.74)	0.004	3.14 (1.62–6.08)	< 0.001

Model adjusted according to Poisson regression with robust variance CMDs = common mental disorders; PPE = personal protective equipment; PR = prevalence ratio.

*Laboratory and X-ray technologists.

## DISCUSSION

We found a higher prevalence of CMDs than reported in other studies conducted during the pandemic. Our study found CMDs in 50% of the participants, whereas the prevalence in Italy was 33.5%.^19^ In addition, studies conducted during other pandemics also found lower rates of CMDs. In Canada, during the SARS outbreak in 2003, the prevalence of CMDs among hospital workers was 29%.^20^ Studies conducted in Brazil prior to the pandemic also reported lower rates of CMDs than the present study. In 2006, a study conducted in the city of Botucatu, São Paulo State, found that 42.6% of HCWs had CMDs.^12^ Since the rate of CMDs observed in this study was higher than in other studies in different contexts, COVID-19 pandemic in Brazil had a strong impact on the mental health of frontline workers. Some reasons for the increase in CMDs during the pandemic include the increased number of cases, overwork, lack of PPE, lack of medication and adequate protocols for treating the virus, and widespread media coverage.^2,3^

The variables associated with an increased risk of CMD were exposure to violence because of being a HCW during the pandemic (including experiencing discrimination, insults, or aggression), age group, and level of confidence in the ability of the workplace to cope with the COVID-19 pandemic. Approximately 73% of participants with CMDs experienced some type of violence during the COVID-19 pandemic. Workplace violence was frequently reported by HCWs and had an impact on their mental health.^5^ It is a complex, multifaceted phenomenon that took on additional precipitating components during COVID-19 pandemic.^21^

Discrimination and violence against HCWs have increased during the COVID-19 pandemic. According to the International Committee of the Red Cross (ICRC), 611 violent incidents were recorded between February and July 2020, 67% of which targeted HCWs worldwide.^21^ These violent incidents occurred primarily in low-income countries, such as India, which experienced a high frequency of such incidents.^22^ The primary motivations for these acts were fear of contracting the disease from HCWs as well as fear, anxiety, restlessness, and despair.

The violence and discrimination experienced by HCWs causes psychological damage that not only affects their mental health but also promotes the development of mental illnesses such as CMDs, which is characterized by somatic, anxiety, and depressive symptoms^23^ and influences the work quality of HCWs.^11^ A study of health workers in Xi’an and Wuhan, China, found that more than 50% of HCWs experienced some type of psychological distress during the pandemic.^24^

The association between exposure to violence and discrimination and the development of CMDs showed that experiencing discrimination increased the risk of developing CMDs by 2.5-fold, and experiencing violence increased the risk by about 2-fold. The more types of violence respondents were exposed to, the higher their risk of developing CMDs; the risk was 2.28-fold higher for HCWs exposed to two types of violence and 3.14-fold higher for those exposed to three types of violence. Physicians are the health professionals at highest risk of presenting with CMDs.

Physicians, administration staff, and nurses were found to be at increased risk of exposure to violence. A hypothesis is that patients and family members view physicians and nurses as representatives of the government in the health system and blame them for problems related to resource scarcity.^5^ During the COVID-19 pandemic, the collapse of the health system,^25^ including the lack of intensive care unit (ICU) beds, sedatives for intubation, and oxygen, may have motivated violent, aggressive attitudes toward health professionals on the part of patients’ family members,^5^ who blamed HCWs for these problems. In addition, the community viewed HCWs as responsible for the spread of COVID-19.^26^

Violence against HCWs has escalated during the COVID-19 pandemic. Menon et al.^22^ found that the spread of misinformation about COVID-19 increased fear of HCWs as potential sources of infection. In Brazil, the national government contributed to the spread of misinformation about the pandemic.^14,27^ The government discredited evidence-based public health measures and medical practices,^28^ creating a sense of insecurity among the population. This may have contributed to increased violence against HCWs. Exposure to abuse has been associated with higher levels of anxiety and depressive symptoms among HCWs. Studies conducted during the COVID-19 pandemic in other countries, particularly low- and middle-income countries, have found similar experiences among HCWs.^10,29^

Confidence in the workplace to handle the COVID-19 pandemic had a protective effect on the mental health of HCWs. The greater their confidence in the workplace, the lower their risk of developing CMDs. The risk of developing CMDs was more than doubled among HCWs who reported no or low workplace confidence, compared with a risk of 0.43 among those who reported high workplace confidence. These data may be explained by social support from supervisors and colleagues, workplace infrastructure, and the presence of the basic inputs necessary to provide adequate health services in the health unit.

Such factors can be considered protective for the mental health of HCWs, as they indicate better relationships among the health care team, adequate institutional support, and less dissatisfaction among patients and family members with the services provided, which may reduce violent attitudes toward HCWs. The study was conducted in one of the largest urban centers of Brazil—the city of São Paulo, where there is greater investment in health and better health infrastructure than in rural areas and areas far from large urban centers. In such locations, the protective effect of workplace trust may not be as prevalent, and the incidence of HCW exposure to mistreatment may be higher or more underreported than in the present study.

Limitations of the study include the cross-sectional design, which did not allow for causal analysis, and the setting in a large urban center, which limits the applicability of the results to nonurban contexts. The prevalence of exposure to violence may have been underestimated because HCWs may not report episodes because of stigma or shame.^30^

## CONCLUSIONS

Our findings reinforce the need to implement protective measures against the mistreatment of HCWs, as the increase in the rate of CMDs among this group during the pandemic was directly related to exposure to violence. First, the media and social networks should educate the population about the pandemic through awareness and reliable information campaigns that are easily accessible, as misinformation about HCWs made them more vulnerable to violence. Second, since trust in the workplace was found to be a protective factor, health managers should propose strategies to strengthen the bonds between HCWs and their institutions, reduce conflicts, and promote communication.^5^
